# Potential regulatory mechanism of TNF-α/TNFR1/ANXA1 in glioma cells and its role in glioma cell proliferation

**DOI:** 10.1515/biol-2022-0023

**Published:** 2022-03-18

**Authors:** Xiaotian Zhu, Guanhui Shi, Jinbiao Lu, Xin Qian, Donglin Wang

**Affiliations:** Department of Pathology, Medical College, Nantong University, No. 19 Qixiu Road, Nantong 226001, Jiangsu Province, P.R. China; Department of Pathology, Jiangyin People’s Hospital, No. 163, Shoshan Road, Jiangyin 214400, Jiangsu Province, P.R. China

**Keywords:** glioma, ANXA1, TNFR1, TNF-α, proliferation

## Abstract

The purpose of this study was to explore the regulatory mechanism of Annexin A1 (ANXA1) in glioma cells in the inflammatory microenvironment induced by tumour necrosis factor α (TNF-α) and its effects on glioma cell proliferation. CCK-8 analysis demonstrated that TNF-α stimulation promotes rapid growth in glioma cells. Changes in tumour necrosis factor receptor 1 (TNFR1) and ANXA1 expression in glioma cells stimulated with TNF-α were revealed through western blot analysis and immunofluorescence staining. Coimmunoprecipitation analysis revealed that ANXA1 interacts with TNFR1. Moreover, we found that ANXA1 promotes glioma cell growth by activating the p65 and Akt signalling pathways. Finally, immunohistochemistry analysis showed an obvious correlation between ANXA1 expression and Ki-67 in glioma tissues. In summary, our results indicate that the TNF-α/TNFR1/ANXA1 axis regulates the proliferation of glioma cells and that ANXA1 plays a regulatory role in the inflammatory microenvironment.

## 1 Introduction

Glioma, the most common brain tumour, is known for its aggressive biological features and poor prognosis [1]. The five-year survival rate for patients with glioma remains low despite the availability of a variety of treatments, including surgery, radiation, and chemotherapy [2,3]. The prognosis of glioblastoma multiforme (GBM) is even worse: the average survival is less than 12 months after diagnosis. Unfortunately, the molecular mechanism has not been fully elucidated [4], and much progress in treating GBM is needed. The latest evidence indicates that different mechanisms, including epigenetic mechanisms, membrane proteins, and the inflammatory microenvironment, contribute to the occurrence and development of GBM [5].

The tumour microenvironment is the local environment within an organ where tumour cells arise and reside [6]. This microenvironment includes the tumour cells themselves, surrounding fibroblasts, inflammatory cells and other cells, and the cell matrix, microvessels, and infiltrating biological molecules in the vicinity [7]. Tumour necrosis factor α (TNF-α), an important cytokine that mediates many inflammatory reactions, is produced by activated macrophages and tumour cells. TNF-α has been shown to promote the formation and progression of the tumour inflammatory microenvironment [8]. Some evidence indicates that TNF-α is a double-edged sword that may exert dual effects on drug resistance, recurrence, and metastasis of glioma [9]. Tumour necrosis factor receptor 1 (TNFR1; 55 kDa) is one of the TNF-α receptors. TNFR1 is a transmembrane protein, the extracellular domain of which binds to TNF-α [10].

Annexins comprise a family of Ca2+-regulated membrane-binding proteins. Annexin A1 (ANXA1), a 37-kDa annexin protein, is involved in various cellular processes, such as transduction, apoptosis, differentiation, and proliferation [11]. ANXA1, expressed from a gene located on chromosome 9q12–q21.2, is also known as chromobindin-9, lipocortin 1, p35, calpactin II, and PLA2 inhibitory protein [12]. ANXA1 has a conserved C-terminal core domain consisting of four highly helical parallel repeats, each approximately 70 amino acids long [13]. Recent evidence shows that ANXA1 participates in the initiation and development of various cancers [14,15]. Excessive expression of ANXA1 in nonsmall-cell lung cancer promotes its carcinogenesis [16], but it is downregulated in breast cancer [17,18]. ANXA1 expression is low in normal brain tissues and largely distributed in ependymal cells and subependymal astrocytes [19]. However, ANXA1 expression is upregulated in malignant glioma tissues; it is secreted by necrotic glioma cells, and abnormal levels of ANXA1 in the tumour microenvironment can activate formyl peptide receptor 1 to enhance the proliferation and invasion of glioma [20]. FoxM1, a well-studied transcription factor, increases ANXA1 expression and promotes the proliferation, invasion, and angiogenesis of glioma [21].

There have been many studies on the relationship between any TNF-α/TNFR1/ANXA1 combination: absence of TNFR1 in intestinal inflammation enhances ANXA1 activity [22]; upregulation of ANXA1 inhibits maturation of human dendritic cells induced by TNF-α [23]; in preeclampsia, ANXA1 correlates positively with TNFR1 [24]. However, studies on the TNF-α/TNFR1/ANXA1 axis with respect to tumours are rare.

In this study, we sought to assess changes in ANXA1 expression in the inflammatory microenvironment and the clinical significance of these changes.

## 2 Materials and methods

### 2.1 Human specimens

In total, 90 specimens were collected from patients with newly diagnosed glioma at the Affiliated Hospital of Nantong University from 2009 to 2015 with approval by the Human Research Council. The collection of specimens was based on the following inclusion criteria: (1) no radiotherapy, chemotherapy, or hormone treatment before surgery; (2) complete clinicopathological data available; and (3) postoperative pathological confirmation of a glioma diagnosis. All 90 patients agreed to participate in the study.


**Informed consent:** Informed consent has been obtained from all individuals included in this study.
**Ethical approval:** The research related to human use has been complied with all the relevant national regulations, institutional policies, and in accordance with the tenets of the Helsinki Declaration, and has been approved by the authors’ institutional review board or equivalent committee.

### 2.2 Immunohistochemistry (IHC)

We used the general two-step IHC method. Briefly, after specimens were dewaxed, rehydrated, and heated to retrieve antigens, the slices were treated with 3% H_2_O_2_ to block endogenous peroxidase activity. The slices were then incubated overnight with primary antibodies at 4°C, with nonimmune serum serving as a negative control. The next day, the slices were washed with phosphate-buffered saline (PBS) and incubated with secondary antibodies before they were stained with 3,3-diaminobenzidine and haematoxylin, sealed with neutral gum, and dehydrated prior to analysis.

### 2.3 Evaluation of IHC staining

Two independent pathologists blinded to the patient data evaluated the immunostained specimens. The staining intensity was graded in comparison with the control as follows: 0 (no staining); 1 (low staining); 2 (moderate staining), and 3 (high staining). The tumour cell ratio score was as follows: 0 (<1%); 1 (1–10%); 2 (10–50%); 3 (50–75%); and 4 (>75%). The scores were summed and designated as follows: 0–3, low expression and 4–7, high expression.

### 2.4 Antibodies

The following primary antibodies were used in this study: anti-TNFR1 (1:2,000, rabbit, Abcam), anti-ANXA1 (1:1,000, goat, Santa Cruz), anti-Ki-67 (1:50, mouse, Abcam), anti-P65 (1:2,000, rabbit, Cell Signaling Technology), anti-p-P65 (1:1,000, rabbit, Cell Signaling Technology), anti-Akt (1:2,000, rabbit, Cell Signaling Technology), anti-p-Akt (S473) (1:1,000, rabbit, Cell Signaling Technology), anti-p-Akt (T308) (1:1,000, rabbit, Cell Signaling Technology), anti-GAPDH (1:5,000, mouse, Abcam), anti-α-tubulin = (1:5,000, mouse, Abcam), anti-Lamin B (1:2,000, mouse, Santa Cruz), anti-HA (1:5,000, mouse, Cell Signaling Technology), and anti-Flag (1:5,000, rabbit, Cell Signaling Technology).

### 2.5 Western blotting (WB)

We used a Bio–Rad protein assay to determine protein concentrations in extracts obtained from cells and tissues. Proteins were then mixed with 2× loading buffer before they were loaded onto gels, separated by sodium dodecyl sulphate–polyacrylamide gel electrophoresis and transferred to polyvinylidene fluoride membranes. The membranes were incubated with primary antibodies overnight at 4°C after blocking. The next morning, the membranes were washed with PBS and incubated with secondary antibodies at room temperature for 2 h. Finally, protein bands were developed with an electrochemiluminescence detection system after the membranes were washed a final time.

### 2.6 Cell culture

We mainly used U251 MG cells and HEK293T cells from the cell library of the Chinese Academy of Sciences. U251 MG cells were cultured in Dulbecco’s modified Eagle’s medium with 10% foetal bovine serum (FBS). HEK293T cells were cultured in Roswell Park Memorial Institute Medium 1640 (RPMI 1640) with 10% FBS. The cells were placed in a humidified, CO_2_ incubator maintained at 37°C, and the medium was changed every 1–3 days due to growth conditions or research needs.

### 2.7 Coimmunoprecipitation (Co-IP)

Cells were collected with medium-strength lysis buffer and processed to produce lysates; the samples were then precleaned with Protein A/G PLUS-Agarose. The samples were then centrifuged at 800×*g* for 5 min to collect the supernatant, with 50 μL was used as input. The remaining cell lysate was then divided equally into two portions: one was mixed with IgG as a control; the other (IP group) was treated with appropriate primary antibodies (assume X). The samples were placed on a rotator at the proper speed and incubated at 4°C overnight. The next day, the samples were mixed with Protein A/G PLUS-Agarose, rotated for 4 h at 4°C, and centrifuged at 1,000×*g* for 8 min to collect the agarose. The samples were heated at 100°C for 10 min to elute the bound proteins, which were then mixed with 2× loading buffer. The samples were centrifuged at 1,000×*g* for 15 min, and the supernatants were loaded onto gels for electrophoresis.

### 2.8 Immunofluorescence staining (IFC)

Cells were plated on small discs in a 24-well plate. The next day, when the cells grew to an appropriate confluence, we discarded the medium, washed the coverslips with PBS, and fixed the cells with 4% paraformaldehyde. The cells were permeabilized with 0.2% Triton X-100 before they were blocked in 1% bovine serum albumin for 20 min and incubated with primary antibodies at 4°C overnight. After washing with PBS, the coverslips were incubated with secondary antibodies for 1 h at room temperature and with 4′,6-diamidino-2-phenylindole for 1 h to stain nuclei. Finally, the coverslips were placed on slides face down and were viewed under an Olympus immunofluorescence microscope.

### 2.9 Separation of nuclear and cytoplasmic/cell membrane protein extracts

We used a commercial kit (Beyotime Biotechnology, China) to isolate cell proteins. Briefly, when cells reached confluence, we washed them with PBS before treating them with hypotonic lysate to disrupt the cell membrane. The lysates were centrifuged at 1.4 × 10^4^×*g* for 2 min; the precipitate comprised nuclei, whereas the supernatant contained the cytoplasmic proteins and cell membrane. The precipitate was treated with hypertonic lysate and centrifuged, and the supernatant was used as the nuclear extract. An appropriate amount of 2* loading was added, mixed well, and stored at −80°C.

### 2.10 Transfection

Overexpression plasmids for ANXA1 and TNFR1 were purchased from the Public Protein/Plasmid Library and used to transfect HEK293T cells. ANXA1 shRNA sequences were synthesized by the Shanghai GeneChem Company as follows: 5′-AUUCUAUCAGAAGAUGUAU-3′(ShANXA1-1), 5′-CUUGUAUGAAGCAGGAGAA-3′(ShANXA1-2), 5′-AGCGCAAUUUGAUGCUGAU-3′(ShANXA1-3), and 5′-AACCAUCAUUGACAUUCUA-3′(ShANXA1-4). The control shRNA sequence was 5′-CCCCUUUUAAAAGGGGCCC-3′(Sh-NC). They were used to transfect U251 MG cells.

Cells were cultured in antibiotic-free medium, and plasmid was added at 80% confluence. The medium was changed after 6 h, and protein was extracted or the cells were stimulated after 48 h of culture.

### 2.11 Cell proliferation assays

U251 MG cells were seeded in a 96-well plate at a density of 1 × 10^4^ per well in a volume of 100 μL. The orange formazan dye produced by the biological reduction of CCK-8 reagent (Cell Counting Kit-8, Dojindo, Kumamoto, Japan) by intracellular dehydrogenase can be dissolved in tissue culture medium, and the amount of formazan produced is proportional to the number of living cells. Two hours after the CCK-8 reagent was added to wells, we measured absorbance at 490 nm using a microplate reader (Immuno Mini NJ-2300).

### 2.12 Statistical analysis

We used the Spearman correlation coefficient to evaluate the relationship between ANXA1 and Ki-67 by IHC. The chi square (*χ* ^2^) test was used to detect the significance of ANXA1 expression with regard to different clinicopathological variables among the 90 clinical samples. Kaplan–Meier (K–M) curves and the log-rank test were used to analyse the influence of ANXA1 on survival. The Cox proportional hazards regression model was applied to calculate the risk of various clinical factors. The model defines survival outcome and survival time as dependent variables and can simultaneously analyse the impact of multiple factors on survival. The significance of the differences among the groups was analysed by one-way analysis of variance. All experiments were conducted independently at least three times. The SPSS 17.0 software package was used to process the data, and *p* values < 0.05 were considered to be statistically significant.

### 2.13 Datasets

A dataset (mRNAseq_693) from the CGGA (Chinese Glioma Genome Atlas) database (http://www.cgga.org.cn/index.jsp) was used to analyse the correlation between ANXA1 and TNFR1, ANXA1 and Ki-67, and ANXA1 and survival.

## 3 Results

### 3.1 Proliferation changes in glioma cells stimulated with TNF-α and expression changes in TNFR1 and ANXA1 in glioma cells stimulated with TNF-α

**Figure 1:**
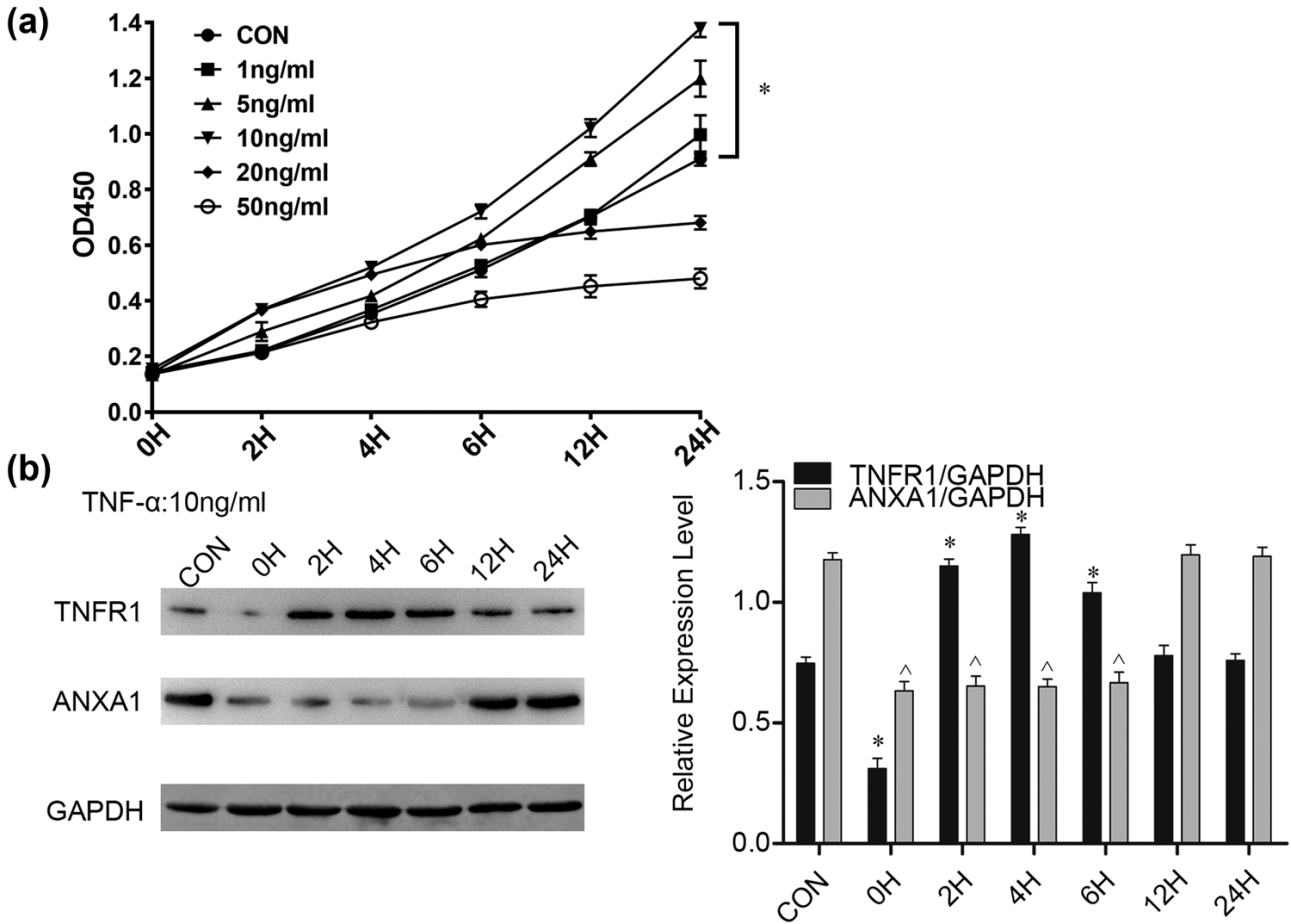
CCK-8 and WB results of glioma cells treated with TNF-α. (a) CCK-8 analysis showed that different concentrations of TNF-α (1, 5, 10, 20, 50 ng/mL) stimulated U251MG cell proliferation within 24 h, and 10 ng/mL TNF-α stimulation achieved the best effect. Cells treated with an equal volume of culture medium served as the control. *n* = 3, *p < 0.05. (b) TNFR1 and ANXA1 expression at different time points in U251 MG cells stimulated with 10 ng/mL TNF-α. Bar charts show TNFR1 and ANXA1 expression compared to that of GAPDH. *, ^*p* < 0.05, compared to the CON group.

To explore the effect of the inflammatory environment on cell proliferation, we treated U251 MG cells with TNF-α (1, 5, 10, 20, and 50 ng/mL) and detected absorbance by CCK-8 analysis at different time points. The fastest proliferation was detected in the 10 ng/mL group (Figure 1a). Next, to determine changes in TNFR1 and ANXA1 expression, we treated U251 MG cells with 10 ng/mL TNF-α and collected them at different time points (0, 2, 4, 6, 12, and 24 h). WB results showed that TNFR1 expression was upregulated at 2, 4, and 6 h. However, ANXA1 expression was downregulated at 0, 2, 4, and 6 h (Figure 1b). As the time of changes in expression of the two proteins coincided, we speculate a relationship between them.

### 3.2 ANXA1 translocates to the nucleus in U251 MG cells stimulated with TNF-α

**Figure 2:**
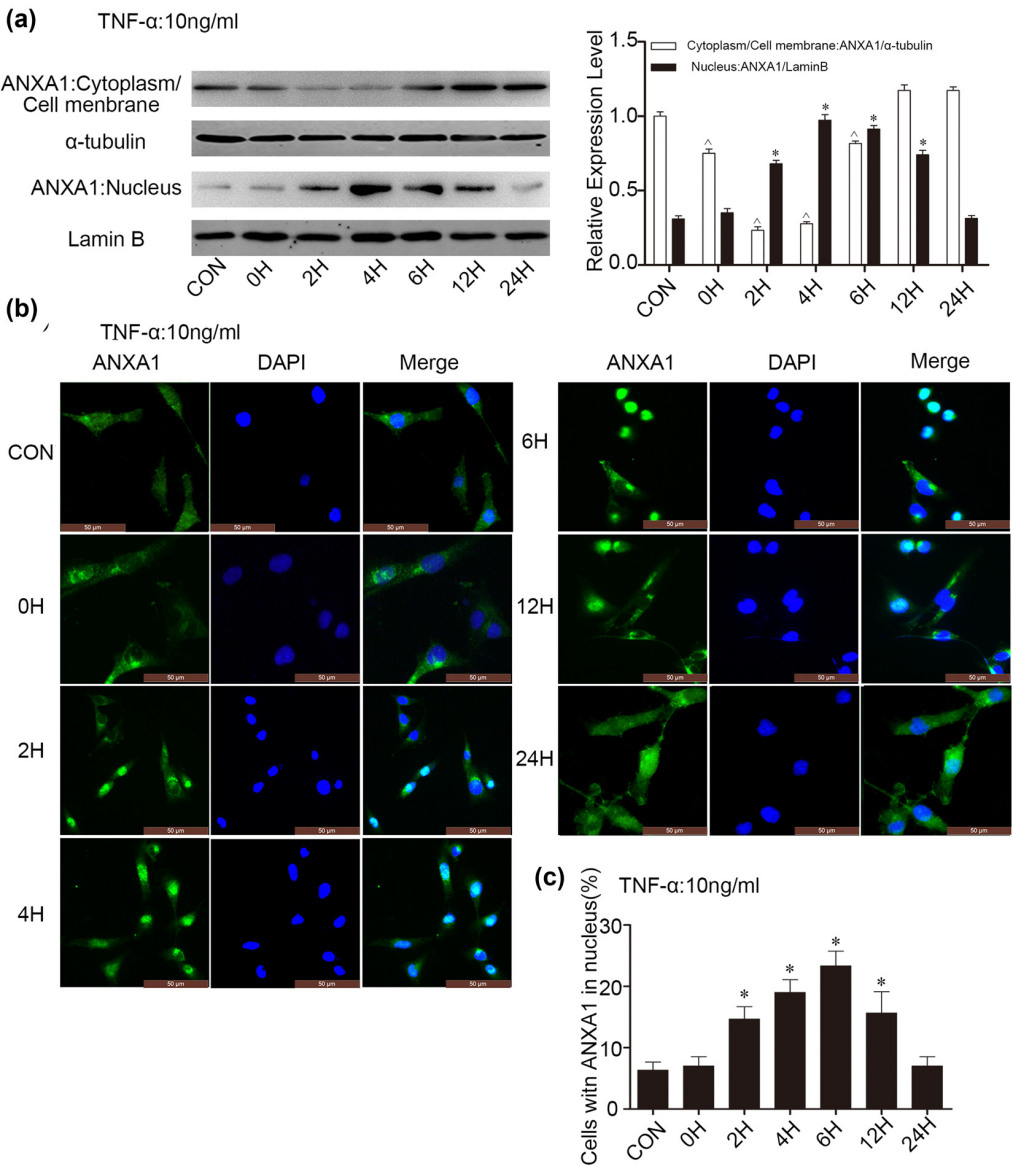
Translocation of ANXA1 in U251 MG cells stimulated with 10 ng/mL TNF-α. (a) WB results of ANXA1 expression in nuclear and cytoplasmic/cell membrane fractions at different time points in U251 MG cells stimulated with TNF-α. White columns show ANXA1 in the cytoplasmic/cell membrane fraction compared to α-tubulin; black columns show ANXA1 in the nuclear fraction compared to Lamin B. *, ^*p* < 0.05, compared to the CON group. (b) IFC of ANXA1 at different time points (SP × 400). (c) Quantitative charts of ANXA1 in the nuclei of U251 MG cells stimulated with TNF-α. *p < 0.05, compared to the CON group.

It has been reported that ANXA1 can localize to the cytoplasm, nucleus, and cell membrane [25]. As its location is not fixed, ANXA1 may have complex functions [26]. Based on this property, we explored whether ANXA1 translocates to the nucleus in glioma cells treated with TNF-α. Based on WB results, ANXA1 levels in the nuclear fraction was obviously increased at 2, 4, and 6 h after treatment, whereas it was dramatically reduced in the cytoplasm/cell membrane fraction at these time points (Figure 2a). We next validated the results by IFC. There was more ANXA1 present in the nucleus at 2, 4, and 6 h after treatment, and levels in the cytoplasm/cell membrane fraction returned to baseline at 24 h (Figure 2b and c).

### 3.3 Interaction between ANXA1 and TNFR1

**Figure 3:**
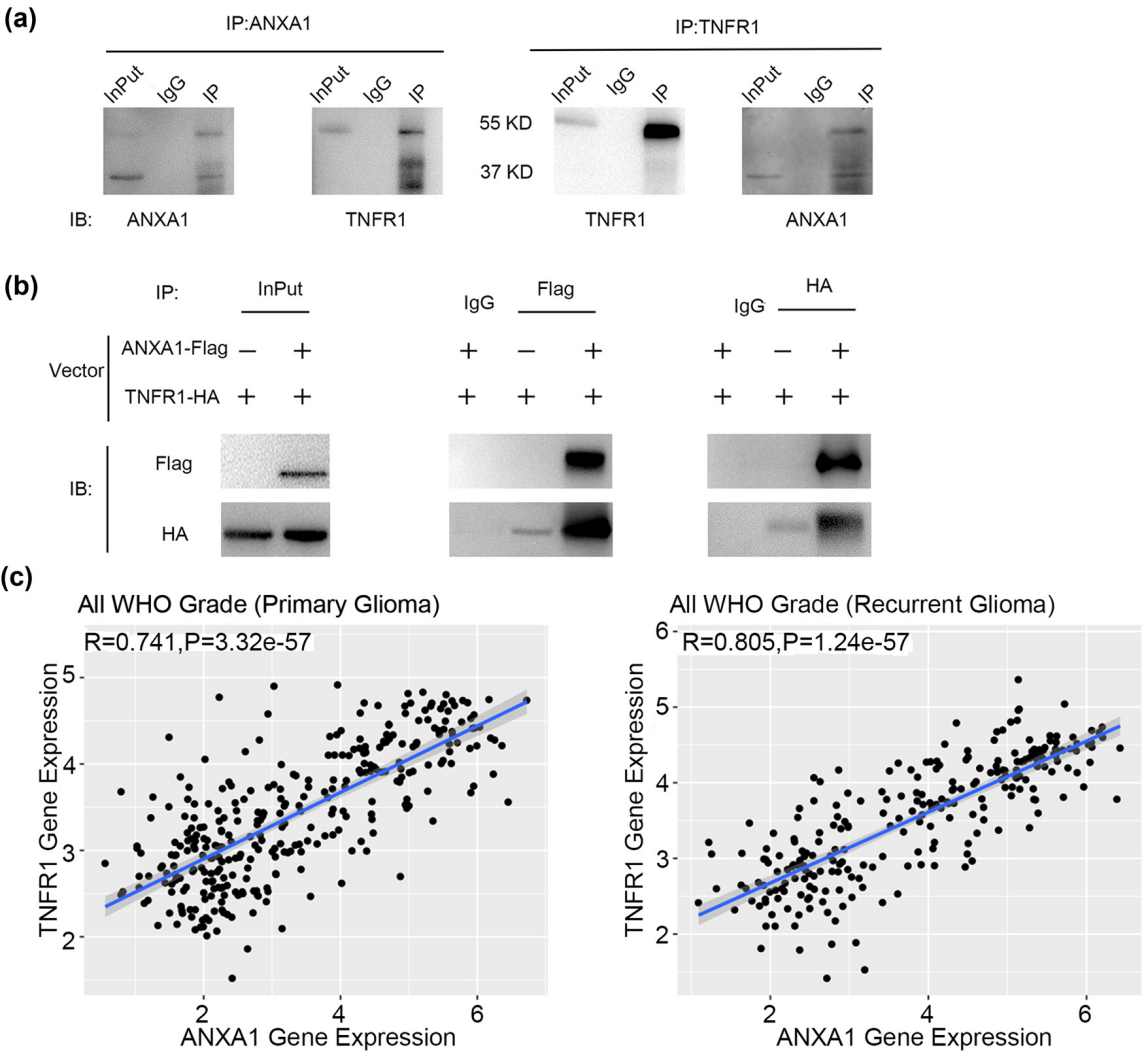
Relationship between ANXA1 and TNFR1. (a) Endogenous co-IP of TNFR1 and ANXA1 in U251 MG cells. The left picture shows the addition of ANXA1 antibody to the cell lysate, and the immunoblotting of the precipitated protein shows TNFR1 positivity. The right picture shows the addition of TNFR1 antibody to the cell lysate, and the immunoblotting of the precipitated protein shows ANXA1 positivity. (b) Exogenous co-IP of TNFR1 and ANXA1 by transfection of TNFR1-HA and/or ANXA1-Flag overexpression plasmids into HEK293T cells. The input group (left) is the WB of normal cell lysate after transfection of the plasmid. The middle figure shows that the Flag antibody can pull HA. The figure on the right shows that the HA antibody can pull Flag. (c) Correlation between ANXA1 and TNFR1 at the mRNA level was analysed using samples from the CGGA database.

As TNFR1 is a receptor of TNF-α, we explored whether translocation of ANXA1 is related to TNFR1. We used U251 MG cells to perform endogenous co-IP and discovered a direct interaction between TNFR1 and ANXA1 (Figure 3a). Next, eukaryotic expression plasmids for Flag-labelled ANXA1 and HA-labelled TNFR1 were constructed. HEK293T cells were transfected with the ANXA1-Flag plasmid and/or the TNFR1-HA plasmid. Cell proteins were collected and precipitated with an anti-Flag or anti-HA antibody, and expression of ANXA1-Flag or TNFR1-HA was detected by WB. The images shown in Figure 3b clearly reveal an interaction between TNFR1 and ANXA1. For verification, we used samples from the CGGA database to determine the relationship between ANXA1 and TNFR1 (Figure 3c), and a positive correlation between ANXA1 and TNFR1 gene expression was observed in both primary and recurrent glioma samples.

### 3.4 Signalling pathways involved in the effects of TNF-α stimulation on glioma cell proliferation

**Figure 4:**
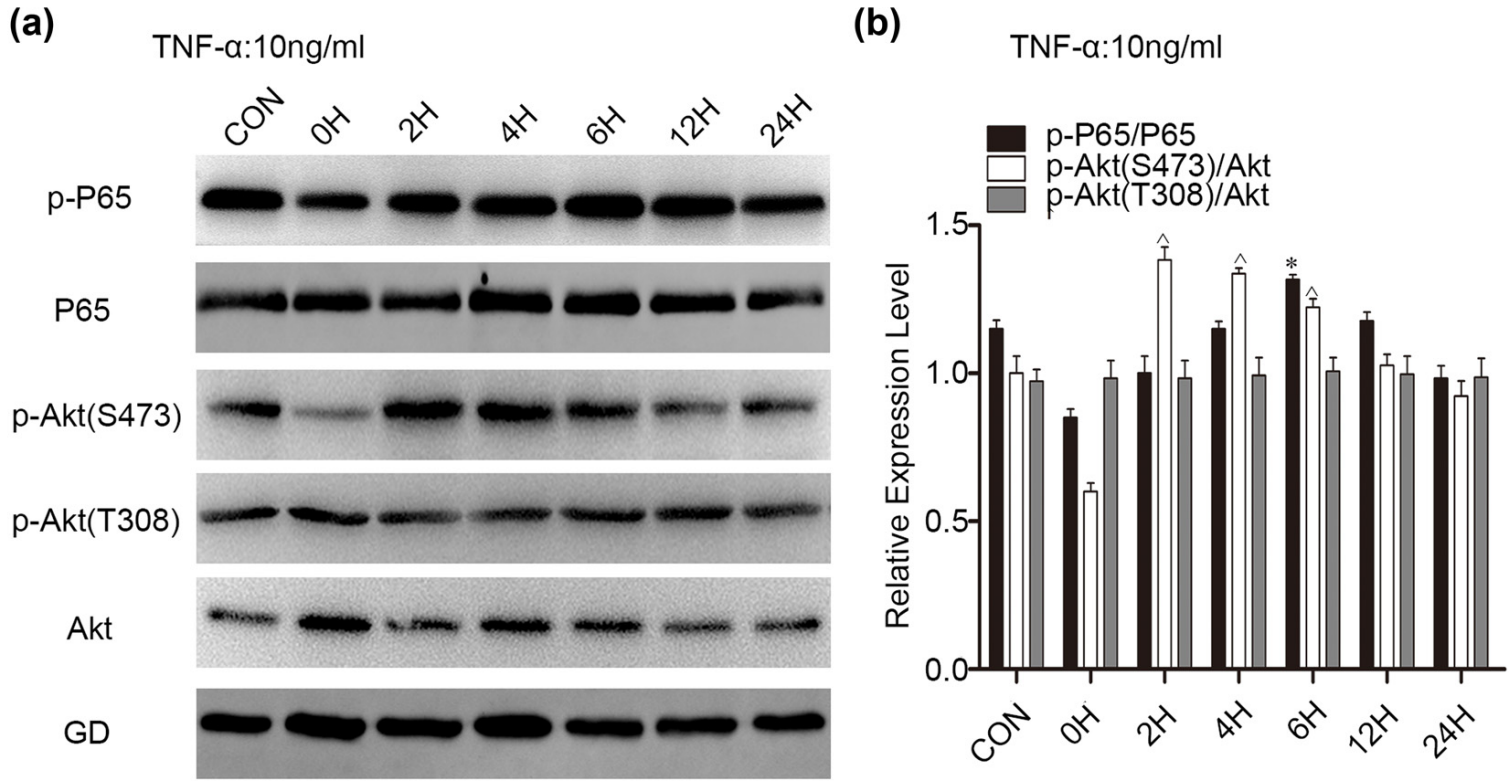
Changes in expression and activation of proteins in the P65 and Akt signalling pathways in U251 MG cells treated with TNF-α. (a) WB results of the P65 and Akt signalling pathways at different time points. (b) Bar charts show p-P65 compared to total P65 and p-Akt (S473) and p-Akt (T308) compared to total Akt. *, ^*p* < 0.05, compared to the CON group.

We explored changes in P65 and Akt, key molecules in two classic carcinogenic pathways, expression and activation upon TNF-α stimulation by WB analysis. NF-κB is a dimeric protein formed by P65 and P50 that has an obvious function of inhibiting apoptosis, and phosphorylation of P65 is evidence of NF-κB activation. Akt, also known as protein kinase B, is an important downstream molecule of PI3K that plays a very important role in regulating cell growth, proliferation, survival, and glucose metabolism and has two important phosphorylation sites: Thr308 and Ser473. Levels of p-P65 were increased at 6 h; those of p-Akt (S473) were increased at 2 h, 4 h, and 6 h. However, no changes were observed in p-Akt (T308) at any time point (Figure 4a and b). This result indicates that TNF-α stimulation may activate P65 and Akt (S473 site).

### 3.5 Effects of silencing ANXA1 on signalling pathways and glioma cell proliferation

**Figure 5:**
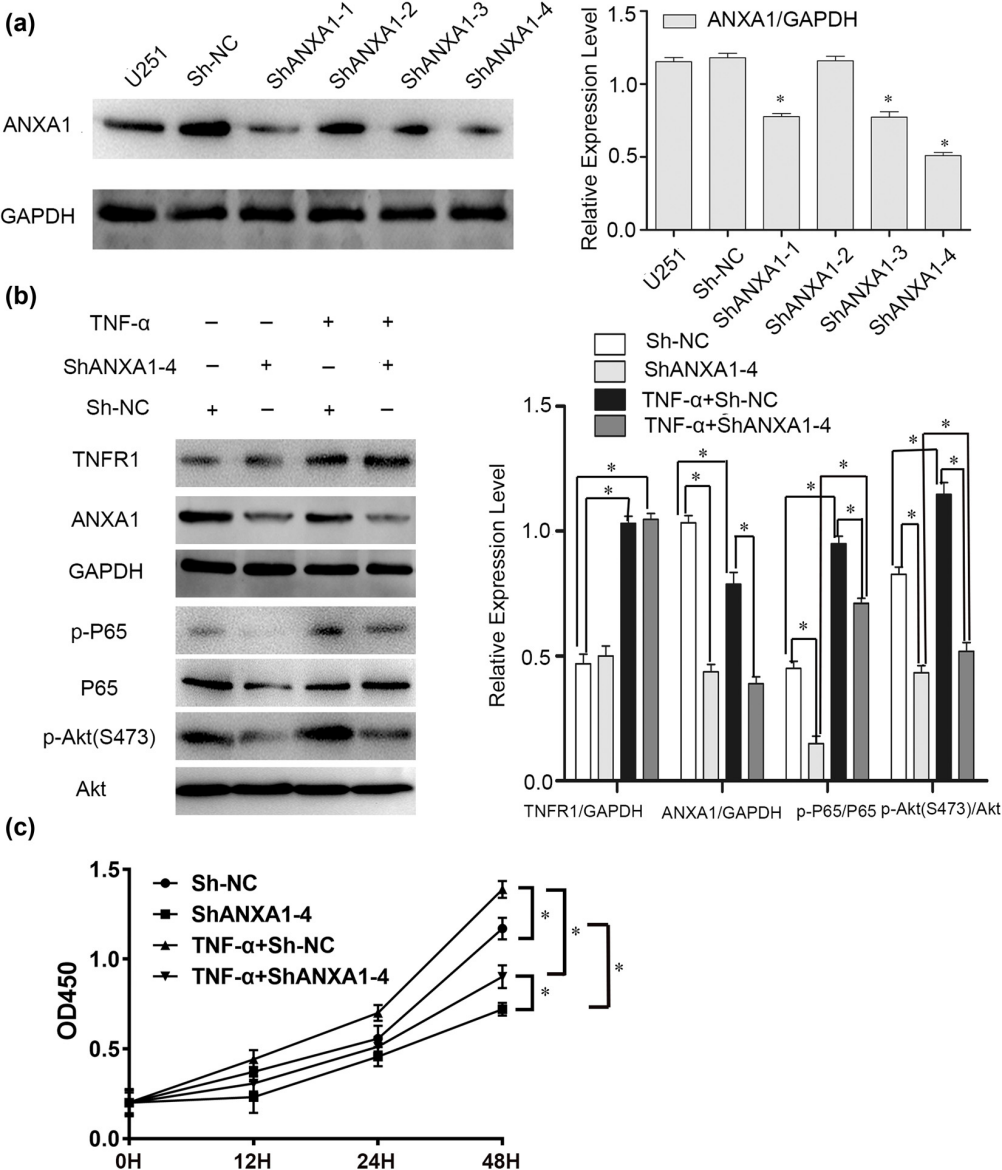
Effects of silencing ANXA1 on expression and activity of proteins in the P65 and Akt signalling pathways and on U251 MG cell proliferation. (a) Knockdown efficiency of the four shRNAs targeting ANXA1 in U251 MG cells. Bar chart shows ANXA1 expression compared to that of GAPDH. *p < 0.05, compared to the Sh-NC group. (b) Changes in TNFR1, ANXA1, P65, and p-Akt (S473) expression levels with different processes in U251 MG cells. Bar chart shows TNFR1 and ANXA1 compared to GAPDH, p-P65 compared to total P65, and p-Akt (S473) compared to total Akt. *p < 0.05. (c) Line chart showing CCK-8 analysis results. *n* = 3, *p < 0.05.

As the P65 and Akt signalling pathways contribute to the proliferation of glioma cells stimulated with TNF-α, we sought to determine whether these signalling pathways are regulated by ANXA1. The knockdown effects of ANXA1 shRNAs are shown in Figure 5a, with ShANXA1-4 resulting in the highest knockdown efficiency. Then, we processed U251 MG cells with the methods illustrated in Figure 5b. Cells were stimulated with 10 ng/mL TNF-α for 6 h, and by comparing the third column (TNF-α + Sh-NC) with the first column (Sh-NC), TNFR1 expression was upregulated after TNF-α treatment but not affected by ShANXA1-4 when comparing the second column (ShANXA1-4) with the first column (Sh-NC). We speculate that ANXA1 is located downstream of TNFR1. Moreover, expression levels of ANXA1, p-P65, and p-Akt (S473) were downregulated in cells transfected with ShANXA1-4 (the second column compared with the first column), and the increased levels of p-P65 and p-Akt (S473) after stimulation with TNF-α were reversed in cells transfected with ShANXA1-4 (the fourth column compared with the second column). Hence, the inhibitory effect of knocking down ANXA1 on the two molecules was rescued by stimulation of TNF-α, further verifying that the two signalling pathways might be regulated by ANXA1 and ANXA1 is downstream of TNFR1. CCK-8 analysis showed U251 MG cell proliferation was downregulated by ShANXA1-4 (cube) but rescued after stimulation with TNF-α (inverted triangle) (Figure 5c).

### 3.6 Upregulated expression of ANXA1 with increasing glioma grade and prognostic significance of ANXA1 in glioma patients

**Figure 6:**
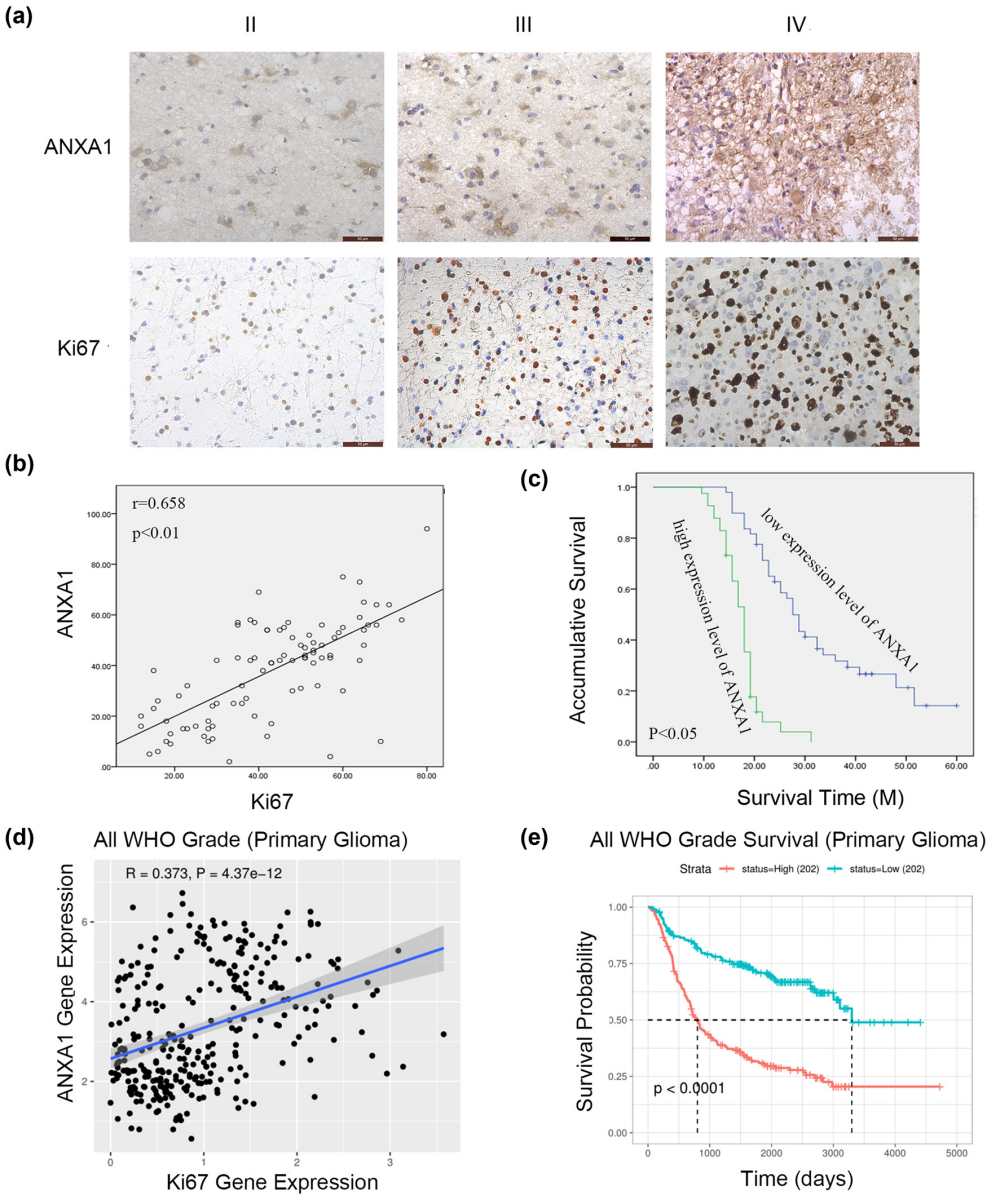
IHC results of ANXA1 expression and univariate analysis of ANXA1 in glioma. (a) Paraffin-embedded glioma tissue sections were stained with anti-ANXA1 antibodies and anti-Ki-67 antibodies followed by counterstaining with haematoxylin (SP × 400). (b) The relationship between ANXA1 and Ki-67 expression by IHC. (c) K–M analysis of ANXA1 in 90 glioma patients. (d) The relationship between ANXA1 and Ki-67 expression based on samples from the CGGA database. (e) K–M analysis of ANXA1 based on samples from the CGGA database.

As the results above suggest that the effects of TNF-α on glioma cell proliferation are mediated by ANXA1, we explored expression of ANXA1 in 90 clinical specimens by IHC, the clinical features of which are shown in Table 1. From the results of the *χ* ^2^ test, ANXA1 expression differed in glioma specimens with different WHO grades. IHC results for ANXA1 and Ki-67 are depicted in Figure 6a. The scattering diagram in Figure 6b shows that expression of ANXA1 positively corresponds with that of Ki-67. We then evaluated the clinical significance of ANXA1. K–M curves and log-rank tests suggested that higher ANXA1 expression indicates poorer survival in glioma (Figure 6c). According to the Cox regression results shown in Table 2, among the many clinical factors that affect prognosis, only WHO grade and ANXA1 expression had a *p* value of less than 0.05. These findings indicate that in addition to WHO grade, ANXA1 expression can be used as an independent prognostic indicator of survival in glioma. Using the CGGA database, the correlation between ANXA1 and Ki-67 was analysed, and univariate survival analysis of ANXA1 at the mRNA level was performed (Figure 6d and e), with the same results as reported above.

**Table 1:** Expression of ANXA1 and clinical pathology characteristics in 90 cases of glioma specimens

Variables	Total	ANXA1 expression		*χ* ^2^ value	*p* value
		Low	High		
Age (years)					
<40	19	10	9	0.032	0.858
≥40	71	39	32		
Gender					
Female	51	26	25	0.569	0.450
Male	39	23	16		
Tumour location					
Frontal	20	11	9	0.146	0.986
Parietal	12	7	5		
Occipital	18	10	8		
Temporal	40	21	19		
Tumour size (cm)					
<4	26	15	11	0.156	0.693
≥4	64	34	30		
WHO grade					
II	33	31	2	32.947	0.000*
III	31	9	22		
IV	26	9	17		
Extent of resection					
Biopsy	34	20	14	1.459	0.482
Total resection	40	19	21		
Subtotal resection	16	10	6		

Pearson’s *χ* ^2^ test for statistical analysis.

*p < 0.05.

**Table 2:** Contribution of various potential prognostic factors to survival through Cox regression analysis of 90 glioma specimens

Characteristics	Hazard ratio	95% CI	*p*-Value
Age	1.157	0.588–2.279	0.673
Gender	1.379	0.794–2.397	0.254
Tumour location	0.883	0.670–1.165	0.380
Tumour size	1.496	0.771–2.903	0.233
WHO grade	5.377	3.325–8.694	0.000*
Extent of resection	0.947	0.719–1.247	0.696
ANXA1 expression	2.670	1.503–4.743	0.001*

CI: confidence interval.

*p < 0.05.

## 4 Discussion

Rapid and invasive tumour growth is associated with the low survival rate of glioma [27], and the tumour inflammatory microenvironment plays an indispensable role in this progression [5]. TNF-α is a vital proinflammatory mediator that plays a central role in the cytokine network [28,29]. To determine the optimal stimulation concentration and time of TNF-α, we evaluated the concentration and time gradient. As shown in Figure 1a, a low concentration of TNF-α promoted cell proliferation; in contrast, a high concentration of TNF-α made cells proliferate slowly and even may led to apoptosis. When the stimulating concentration of TNF-α was 10 ng/mL, cell growth was optimal and increased over time. Therefore, we chose 10 ng/mL TNF-α for experiments. TNFR1, a receptor of TNF-α, recruits a series of related proteins via the TNF receptor-associated death domain that interacts with its cytoplasmic domain to regulate signal transduction and cellular biological functions [30]. ANXA1 has been studied in many tumours in the past 20 years, with a variety of biological functions. ANXA1 is specifically expressed in tumours, which makes it an important biological indicator for early tumour diagnosis and prognosis.

Our experiments identified that expression of TNFR1 and ANXA1 is altered in glioma cells stimulated with TNF-α (Figure 1b). Coincidentally, the timing of the changes coincided. We hypothesize a connection between the two. We then explored the regulatory mechanism of ANXA1 and its role in glioma cell proliferation. It has been demonstrated that nuclear translocation of ANXA1 is involved in neuronal apoptosis after ischaemic stroke [31] and induces retinal ganglion cell apoptosis after ischaemia–reperfusion injury through the p65/IL-1beta pathway [32]. Our research showed that ANXA1 is translocated to the nucleus in glioma cells exposed to TNF-α (Figure 2). Nevertheless, the exact regulatory mechanism responsible remains unclear. Next, we performed co-IP in U251MG cells (Figure 3a, endogenous) and HEK293T cells (Figure 3b, exogenous). HEK293T cells rarely express the endogenous receptors required for extracellular ligands and are relatively easy to transfect; it is a very commonly used cell line for expressing and studying exogenous genes. Based on the results shown in Figure 3, we hypothesized that translocation of ANXA1 to the nucleus is related to its interaction with TNFR1.

TNF-α is involved in activation of the NF-kB and PI3K-Akt signalling pathways [33,34], and ANXA1 regulates the proliferation, migration, and invasion of glioma cells via PI3K/AKT signalling [35]. We investigated P56 and Akt, the key molecules of the two pathways. The data in Figure 4 show that the two pathways are involved in the proliferation of glioma cells stimulated with TNF-α and that these two signalling pathways might be regulated by ANXA1 (Figure 5b). In addition, knockdown of ANXA1 slowed glioma cell proliferation (Figure 5c). All these results indicate that ANXA1 contributes to glioma cell proliferation upon TNF-α stimulation. In other words, TNF-α promotes cell proliferation via the TNF-α/TNFR1/ANXA1 axis. Finally, we detected ANXA1 expression in primary clinical specimens and samples from the CGGA database and found that ANXA1 correlated positively with Ki-67 and was highly expressed in high-grade gliomas. According to data analysis, ANXA1 is a potential prognostic factor of gliomas, consistent with the literature. ANXA1 is useful as a prognostic biomarker in lower grade glioma patients with MGMT promoter methylation [36] and a prognostic indicator and an immunotherapy marker for the tumour microenvironment in glioma [37].

Although the main treatment for glioma is maximal safe resection with radiotherapy and chemotherapy [38,39], there have been remarkable advances in cancer immunotherapy since 2015, and multiple cancer types now benefit from these immunotherapies. However, as glioma immunotherapy still faces challenges [40], it is of great significance to discover new glioma biomarkers and study their internal mechanisms. Our research will help in the exploration of new immunotherapy mechanisms and lay the foundation for molecular targeted therapy of glioma. In general, the effect of ANXA1 on tumour cells seems to be diverse and sometimes even opposing due to mutations in the gene, hypermethylation of the promoter and subsequent loss of transcription, posttranslational modification of the protein, and defects in protein storage, among others [14]. When targeting ANXA1 for treatment or research, it is necessary to have a global view. Furthermore, it is necessary to consider its mechanism from multiple angles when conducting research on an organ, tissue, or cell.

## 5 Conclusion

In summary, our study elaborates on the possible mechanism of ANXA1 in the inflammatory microenvironment of glioma cells upon TNF-α stimulation and the role of ANXA1 in glioma cell proliferation. In addition, we analysed the clinical significance of ANXA1 and found its expression to be an independent risk factor for glioma.
